# Grapevine leafroll-associated virus 3

**DOI:** 10.3389/fmicb.2013.00082

**Published:** 2013-04-16

**Authors:** Hans J. Maree, Rodrigo P. P. Almeida, Rachelle Bester, Kar Mun Chooi, Daniel Cohen, Valerian V. Dolja, Marc F. Fuchs, Deborah A. Golino, Anna E. C. Jooste, Giovanni P. Martelli, Rayapati A. Naidu, Adib Rowhani, Pasquale Saldarelli, Johan T. Burger

**Affiliations:** ^1^Department of Genetics, Stellenbosch UniversityStellenbosch, South Africa; ^2^Biotechnology Platform, Agricultural Research CouncilStellenbosch, South Africa; ^3^Department of Environmental Science, Policy and Management, University of CaliforniaBerkeley, CA, USA; ^4^School of Biological Sciences, University of AucklandAuckland, New Zealand; ^5^The New Zealand Institute for Plant and Food ResearchAuckland, New Zealand; ^6^Department of Botany and Plant Pathology, Oregon State UniversityCorvallis, OR, USA; ^7^Department of Plant Pathology and Plant-Microbe Biology, Cornell UniversityGeneva, NY, USA; ^8^Department of Plant Pathology, University of CaliforniaDavis, CA, USA; ^9^Plant Protection Research Institute, Agricultural Research CouncilPretoria, South Africa; ^10^Department of Soil, Plant and Food Sciences, University Aldo Moro of BariBari, Italy; ^11^Department of Plant Pathology, Irrigated Agriculture Research and Extension Center, Washington State UniversityProsser, WA, USA; ^12^Institute of Plant Virology, National Research CouncilBari, Italy

**Keywords:** grapevine leafroll disease, GLRaV-3, ampelovirus, *Closteroviridae*, genetic variants

## Abstract

Grapevine leafroll disease (GLD) is one of the most important grapevine viral diseases affecting grapevines worldwide. The impact on vine health, crop yield, and quality is difficult to assess due to a high number of variables, but significant economic losses are consistently reported over the lifespan of a vineyard if intervention strategies are not implemented. Several viruses from the family *Closteroviridae* are associated with GLD. However, *Grapevine leafroll-associated virus 3* (GLRaV-3), the type species for the genus *Ampelovirus*, is regarded as the most important causative agent. Here we provide a general overview on various aspects of GLRaV-3, with an emphasis on the latest advances in the characterization of the genome. The full genome of several isolates have recently been sequenced and annotated, revealing the existence of several genetic variants. The classification of these variants, based on their genome sequence, will be discussed and a guideline is presented to facilitate future comparative studies. The characterization of sgRNAs produced during the infection cycle of GLRaV-3 has given some insight into the replication strategy and the putative functionality of the ORFs. The latest nucleotide sequence based molecular diagnostic techniques were shown to be more sensitive than conventional serological assays and although ELISA is not as sensitive it remains valuable for high-throughput screening and complementary to molecular diagnostics. The application of next-generation sequencing is proving to be a valuable tool to study the complexity of viral infection as well as plant pathogen interaction. Next-generation sequencing data can provide information regarding disease complexes, variants of viral species, and abundance of particular viruses. This information can be used to develop more accurate diagnostic assays. Reliable virus screening in support of robust grapevine certification programs remains the cornerstone of GLD management.

## Introduction

Grapevine leafroll disease (GLD) is one of the most important viral diseases affecting wine, juice, and table grape cultivars, as well as rootstocks. It currently ranks as one of the most important diseases affecting wine grape cultivars, comparable with several fungal diseases (Naidu et al., 2008). While it is generally accepted that the etiology of GLD is complex, with a number of closteroviruses associated with the disease, it is *Grapevine leafroll-associated virus 3* (GLRaV-3) in the genus *Ampelovirus* that carries the mantle as the “main etiological agent” contributing to the disease.

Here, we review the current state of knowledge on this ubiquitous pathogen. The review is comprehensive in covering most aspects of GLRaV-3 research, but pays special attention to the more recent molecular characterization. The virus genome organization and gene functions of the 13 ORFs (12 ORFs in the case of group VI variants), which are based on the comparative sequence analysis, the expression of the encoded proteins and the replication of the genome are discussed. The genetic variability between GLRaV-3 isolates is addressed in detail, and a proposal is made to standardize the naming of the genetic variant groups identified to date. A summary of diagnostic assays employed to detect the virus is also presented, with a special emphasis on the application of next-generation sequencing technologies and its potential to revolutionize our understanding of the metagenomic nature of virus infections in grapevine varieties. The review is concluded with a discussion of the various levels of host-pathogen interactions, highlighting a very intriguing potential role of small RNAs (sRNAs) in this complex plant virus interplay.

## A Historical Outline of Grapevine Leafroll Disease

Grapevine leafroll disease is one of the major virus diseases of grapevine (*Vitis vinifera* L.) that may have originated in the “Old World,” from where it spread, primarily through commercial trading of propagation material, to attain its current worldwide geographical distribution. Evidence that leafroll occurred in Europe and in other regions of the Mediterranean basin and Near East before the introduction of phylloxera (*Dactulosphaira vitifoliae*) from the eastern United States in the mid nineteenth century (Gale, 2002), rests on a number of observations: (i) Description in the old European literature of “reddening,” an abnormal condition of red-berried grapevine cultivars consisting of early discoloration of the leaves, which accumulated carbohydrates, and showed downward rolling of the laminae. This condition was frequently attributed to physiological disorders and referred to as “rougeau” in France (Ravaz and Roos, 1905; Pacottet, 1906) and “rossore” in Italy (Arcangeli, 1907). (ii) Presence of dried grapevine shoots in a herbarium, collected in north-eastern Sicily between 1880 and 1888, that display various degrees of leaf discoloration (Martelli and Piro, 1975). The leaves of one specimen, in particular, were black, thicker and heavier than normal, and brittle, as indicated by their extensively cracked surface. This and other specimens were labeled “*Malattie della vite. Rossore. Foglie quasi nere o rosso-nere. Vitigno nero. Settembre 1885–1886*” (Grapevine diseases. Reddening. Leaves almost black or reddish-black. Red-berried cultivar. September 1885–1886). (iii) Presence of GLD-infected vines in an abandoned vineyard established between 1889 and 1891 by the University of California as a varietal test block in a secluded locality at the foothills of the Sierra Nevada Mountains in Amador County (California), prior to the wide use of rootstocks made necessary by the spread of phylloxera around 1900 (Luhn and Goheen, 1970). (iv) Occurrence of some of the leafroll-associated viruses, especially *Grapevine leafroll-associated virus 1* (GLRaV-1) and *Grapevine leafroll-associated virus 3* (GLRaV-3) in own-rooted vines from countries where grapes have been grown for centuries, e.g., Cyprus (Ioannou, 1993), Yemen (Martelli et al., 1994), parts of China (Pio Ribeiro et al., 2004), Armenia, and southern Turkey (P. La Notte and G. P. Martelli, pers. comm.), which are still phylloxera-free.

The etiology of GLD remained undetermined until the successful transmission by grafting from grape to grape provided evidence of its infectious nature (Scheu, 1935). Although it was established that GLD was an infectious disease of possible viral origin, its causal agent was still unknown. The breakthrough came in the late 1970s when Namba et al. (1979) found closterovirus-like particles in thin sections of phloem tissue and in leaf dips from GLD affected vines, suggesting that this type of viruses could be the disease agent. This finding was soon confirmed by ultrastructure studies of leaf tissues of GLD affected vines (Faoro et al., 1981; Castellano et al., 1983).

A second breakthrough came when Gugerli et al. (1984) identified two serologically unrelated closterovirus-like viruses with particle length of 2,200 and 1,800 nm, respectively, in purified preparations from symptomatic grapevine leaves. These two viruses were denoted grapevine leafroll-associated viruses 1 and 2 (GLRaV-1 and GLRaV-2). A third serologically unrelated species, that was speculated (Rosciglione and Gugerli, 1986) and then proven to be transmitted by pseudococcid mealybugs (Rosciglione and Gugerli, 1987), was added when the nomenclature of viruses associated with GLD was revised (Boscia et al., 1995). Subsequent studies in Europe (Gugerli et al., 1984; Rosciglione and Gugerli, 1986; Zimmermann et al., 1990) and the USA (Hu et al., 1990) have identified five serologically unrelated clostero-like viruses associated with the GLD complex. The introduction of molecular technologies, and especially nucleotide sequencing, increased the number of closteroviruses associated with leafroll disease to over10, before a sensible consolidation was proposed, reducing the number to five (Martelli et al., 2012). Nonetheless, GLRaV-3 remains the uncontested primary agent associated with GLD. Milestones in GLRaV-3 research in recent years include the production of antibodies and subsequent development of diagnostic assays; sequencing of the genome; transmission and metagenomics-based epidemiological studies, confirming GLRaV-3 as the major causative agent of GLD. Details of these aspects are discussed below.

## Grapevine Leafroll Disease

### Symptomatology

**Figure 1:**
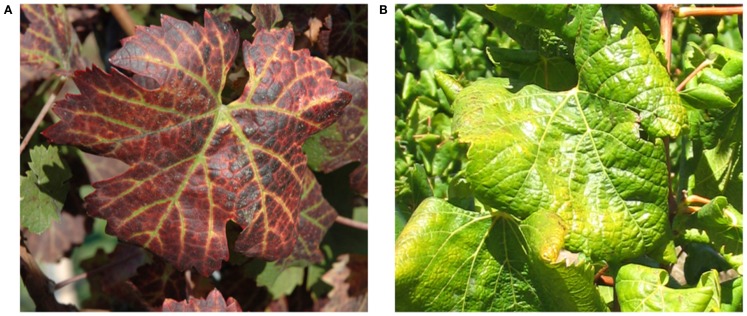
**Typical leafroll diseased vines: (A) a red cultivar, *Vitis vinifera* cv Cabernet Franc; (B) a white cultivar, *Vitis vinifera* cv Chardonnay**.

Symptoms of GLD can vary greatly with the season, grape cultivar, and climatic conditions. Additionally, some varieties can be completely symptomless, like some rootstocks and certain white *V. vinifera* cultivars, which can serve as a reservoir from where GLD can be transmitted to cultivars that would display a range of symptoms. In spring, bud break and shoot development is often delayed in GLD affected vines. This is usually a short-lived phenomenon, lasting for only a few weeks. Leaf symptoms first become apparent in early to mid-summer, often appearing earlier on vines which are water stressed. These symptoms increase in number and severity until late autumn (Figure 1). In most red cultivars, GLD causes reddening of the interveinal areas while the primary and secondary veins remain green. Leaves of some red cultivars, particularly those with deeply pigmented fruit, develop uniform red color without green veins. In white cultivars, the interveinal area may become chlorotic. This symptom is often subtle and may not be recognizable. In late autumn the leaf margins roll downward however, the extent of leaf-rolling varies considerably among infected cultivars. White cultivars, like Chardonnay, show pronounced leaf-rolling by harvest time (Figure 1), while Thompson Seedless and Sauvignon Blanc, show little or no leaf-rolling at all. In these white cultivars, GLD is nearly impossible to detect visually. As the growing season progresses, more and more leaves display GLD symptoms, progressing from the base of the shoot to the tip. American rootstocks are usually asymptomatic carriers of the associated viruses except for a variable decrease in vigor. Hence, the risk of disseminating the disease is great if untested rootstocks are used for propagation and grafting (Weber et al., 1993; Martelli and Boudon-Padieu, 2006; Martelli et al., 2012).

### Associated viruses

To date a number of different viruses in the family *Closteroviridae* have been reported to be associated with GLD. These viruses include *Grapevine leafroll-associated viruses* (GLRaV) 1–9 and a group of more recently described viruses (GLRaV-Pr, GLRaV-De, and GLRaV-Car). All these viruses belong to the genus *Ampelovirus* except for GLRaV-2, which is in the genus *Closterovirus*, and GLRaV-7, which is in the tentative genus *Velarivirus* (Al Rwahnih et al., 2011) (Table 1). GLRaV-8 sequences (GenBank: AF233936) are not of viral origin, but rather similar to the sequences of the *V. vinifera* host; therefore, GLRaV-8 is no longer recognized as a virus species (Bertsch et al., 2009; Martelli et al., 2012). GLRaV-1, -3, and most strains of -2 usually induce stronger leaf symptoms compared to other leafroll-associated viruses. All known isolates of GLRaV-7 show very mild or uncertain GLD symptoms.

**Table 1:** **Current classification and some properties of Grapevine leafroll-associated viruses (GLRaVs)**.

Virus	Genus	Coat protein (kDa)	Genome size (nt)	GenBank access. No.	ORFs	Vectors	First record *fide* [Boscia et al. (1995), Martelli et al. (2012)]
GLRaV-1	Ampelovirus	34	18659	JQ023131	9	Mealybugs and soft scale insects	Gugerli et al. (1984)
GLRaV-2	Closterovirus	22	16494	AY88162	8	Unknown	Zimmermann et al. (1990)
GLRaV-3	Ampelovirus	35	18498	EU259806	12	Mealybugs, soft scale and scale insects	Zee et al. (1987)
GLRaV-4	Ampelovirus	35	13830	FJ467503	6	Mealybugs	Hu et al. (1990)
GLRaV-5^a^	Ampelovirus	35	13384^b^	FR822696	6	Mealybugs	Walter and Zimmermann (1991), Zimmermann et al. (1990)
GLRaV-6^a^	Ampelovirus	35	13807	FJ467504	6	Mealybugs	Gugerli and Ramel (1993), Gugerli et al. (1997)
GLRaV-7	Velarivirus^c^	37	16496	HE588185	10	Unknown	Choueiri et al. (1996)
GLRaV-9^a^	Ampelovirus	35	12588^b^	AY29781	6	Mealybugs	Alkowni et al. (2004)
GLRaV-Pr^a^	Ampelovirus	30	13696	AM182328	6	Mealybugs	Maliogka et al. (2009)
GLRaV-Car^a^	Ampelovirus	29	13626	FJ907331	6	Unknown	Abou Ghanem-Sabanadzovic et al. (2010)

*^a^Future classification might list these as strains of GLRaV-4*.

*^b^Nearly complete sequence*.

*^c^Tentative classification*.

**Figure 2:**
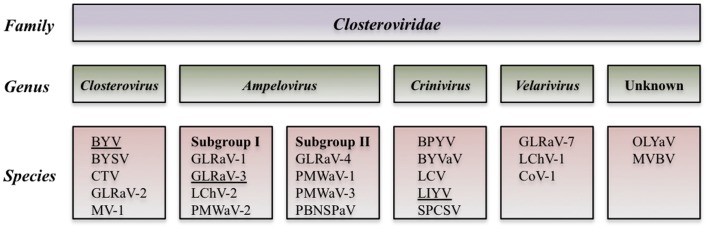
**Diagram of the proposed taxonomic modification that is in process to be examined by the International Committee of Taxonomy of Viruses (Martelli et al., 2012)**.

Analysis of the biological and molecular criteria of GLRaVs in the genus *Ampelovirus* suggested that these viruses belong to two different subgroups: subgroup I includes GLRaV-1 and GLRaV-3 along with *Pineapple mealybug wilt-associated virus 2* (PMWaV-2) and *Little cherry virus 2* (LChV-2). Subgroup II includes GLRaV-4 plus PMWaV-1, PMWaV-3, and *Plum bark necrosis stem pitting-associated virus* (PBNSPaV). Further biological, serological and molecular data showed that GLRaV-4, -5, -6, -9, -Pr,-De, and -Car are closely related and all could be considered as different strains of GLRaV-4 (Martelli et al., 2012). The proposed taxonomic modification is in process to be examined by the International Committee on Taxonomy of Viruses (ICTV) (Figure 2).

### Geographical distribution

Grapevine leafroll disease has a significant impact on grape-growing regions worldwide, resulting in significant losses. Among all viruses associated with GLD, GLRaV-3 is by far the most noticeable and widely distributed in different regions of the world, including Europe, South and North America, Middle East, Northern and Southern Africa, Asia, and Oceania (Pio Ribeiro et al., 2004; Martin et al., 2005; Cabaleiro and Segura, 2006; Charles et al., 2006a; Pietersen, 2006; Sharma et al., 2011). Evidently, this virus has been introduced to most grape growing regions by exchange and propagation of infected plant material and subsequent local spread by vegetative propagation and insect vectors (Cabaleiro and Segura, 2006; Martelli and Boudon-Padieu, 2006; Sharma et al., 2011; Tsai et al., 2012).

### Resistant grapevine varieties

Severity of symptoms and yield losses due to GLD depend on the combination of viruses, cultivars, rootstocks, climate, soil, and viticultural practices. Although some varieties are asymptomatic no sources of GLD resistance have yet been found in *V. vinifera* cultivars and clones (Weber et al., 1993; Martelli, 2000).

Responses to infection by different GLRaVs, or combinations of these, by different grape rootstocks vary significantly. For example, it has been observed that grapevines propagated on the rootstocks Freedom and Harmony were severely affected by these viruses, in contrast to those grafted on AxR, which remained unaffected (Golino et al., 2003). The RG and PN strains of GLRaV-2 have been reported to cause lethal graft incompatibility in certain scion and rootstock combinations. The combination of *V. vinifera* and rootstocks: Couderc 1616, Kober 5BB, Teleki 5C, Couderc 3309, and Paulsen 1103 were shown to be most sensitive (Bertazzon et al., 2010; Alkowni et al., 2011). The cause of this lethal effect remains to be elucidated.

### Disease management

Clean stock and certification programs have been established in several countries in order to produce, maintain, and distribute healthy grapevines. These programs test for GLD and other diseases for the maintenance and production of clean stocks. These clean stocks can be generated by virus elimination strategies that include heat therapy, meristem tip culture (Savino et al., 1991), somatic embryogenesis (Gambino et al., 2006), and even chemotherapy of *in vitro*-grown explants (Panattoni et al., 2007). As a disease management strategy growers are currently advised to plant certified material derived from virus-tested stocks when establishing new vineyards. In areas where this is not possible due to winemaker preferences or other factors, propagating stocks should be carefully screened for viruses using rigorous laboratory tests. Maximizing the distance between new plantings and virus-infected old plantings should reduce the rate of spread. Roguing of infected vines diagnosed with the GLD associated viruses should also reduce spread if done once symptoms are present, especially in new plantings. It may be helpful to minimize the movement of farm equipment between vineyards since this practice may assist mealybug dispersal in vineyards. The use of pesticide sprays to control the mealybug vectors of leafroll may be useful in regional control programs but are not always effective in controlling spread (Golino et al., 2002, 2008; Pietersen et al., 2009). Disease management practices currently used in different world regions are discussed in more detail elsewhere in this research topic (Almeida et al., 2013).

## Taxonomy and Physical Properties of GLRaV-3

**Figure 3:**
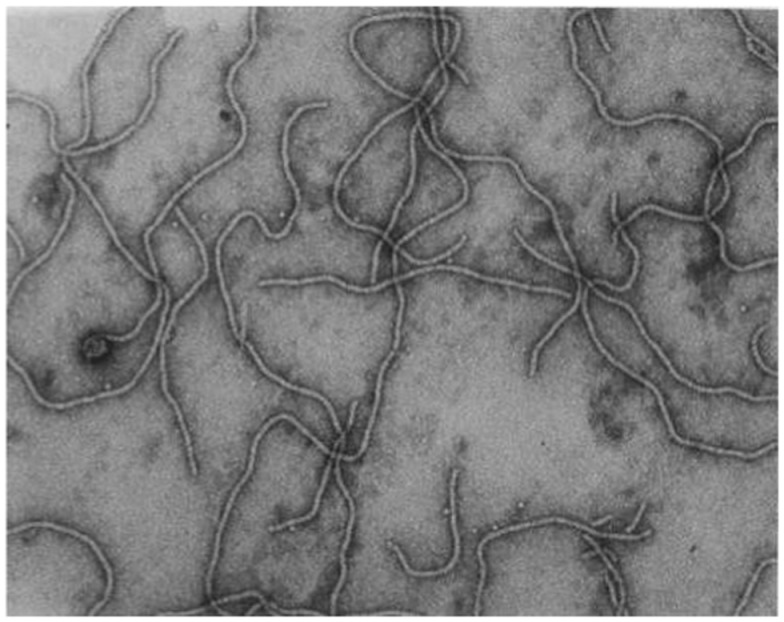
**Transmission electron micrograph of negatively stained, purified GLRaV-3 particles, using 1% (w/v) aqueous uranyl acetate staining**. Picture taken by G. G. F. Kasdorf.

*Grapevine leafroll-associated virus 3* is the type species of the genus *Ampelovirus*, family *Closteroviridae*, and a member of the proposed subgroup I of this genus (Martelli et al., 2011, 2012). GLRaV-3 particles are flexuous filaments, 1,800 × 12 nm in size, showing distinct cross banding (Figure 3). They are helically constructed and contain approximately 10 protein subunits per turn of the helix, which has a pitch of about 3.5 nm (Martelli et al., 2011). The 34 kDa major coat protein (CP) coats the whole length of the virions, except for 5′ extremity (*ca*. 100 nm). The 5′ end of the viral genome is likely to be encapsidated by the virion tail structure, similar to that of other members of the family *Closteroviridae*, which comprises proteins coded for by ORF4 (HSP70h), ORF5 (p55), and ORF7 (CPm) of the viral genome, and might be instrumental in determining cell-to-cell and systemic transport (Dolja et al., 2006). However, no research has been conducted on the composition of a virion tail/head structure at the 5′ extremity of the GLRaV-3 virion; and the proteins associated with such a structure are inferred from homologous proteins for other viruses (e.g., BYV and CTV). The reference to a virion tail at the 5′ end of the genome is suboptimal and should ideally be referred to as the virion head, as suggested in the ninth report of the International Committee on Virus Taxonomy (ICTV) (2009). To avoid confusion, and to be in line with published data on BYV and CTV, the virion structure at the 5′ extremity will be referred to as the virion tail in this review. The genome is a single-stranded, positive-sense RNA molecule constituting *ca*. 5% of the particle weight. Its 5′ end is likely to be capped and the 3′ end is not polyadenylated.

## GLRaV-3 Genome Organization and Functions of Encoded Proteins

GLRaV-3 has a mono-partite, positive-strand RNA genome of ∼18,500 nucleotides. The first complete, 18,498 nucleotide-long, genome sequence of GLRaV-3 was determined for isolate GP18 from South Africa (Maree et al., 2008). This genome has a 737 nucleotide-long 5′UTR with a very high uracil content (48.5%) (Maree et al., 2008). The large size and U-rich composition of the GLRaV-3 5′UTR are unusual features among members of the family *Closteroviridae* and likely explain technical problems that resulted in the incomplete 5′UTR sequence presented by Ling et al. (2004) and Engel et al. (2008). This issue was unequivocally resolved in the following work by using 5′RACE for the molecular cloning of the 5′-proximal part of the GLRaV-3 genome (Maree et al., 2008; Jarugula et al., 2010; Jooste et al., 2010).

**Figure 4:**
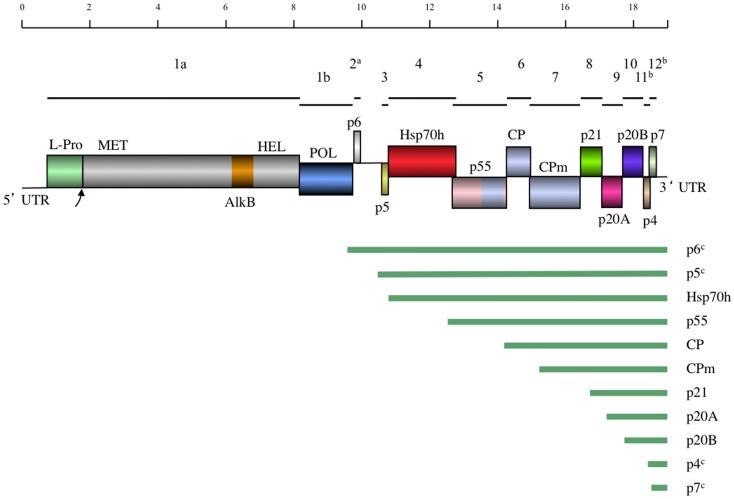
**A schematic diagram of the GLRaV-3 genome to scale**. Lines above the genome map indicate the positions of the ORFs and their respective corresponding numbers. In the genome map, boxes indicate positions of genes with gene products and domains indicated. Homology between the CP and p55 and CPm is indicated by the same coloring. UTR, Untranslated region; L-Pro, Leader papain-like protease; MET, Methyltransferase; AlkB, AlkB domain; [Fe (II)/2-oxoglutarate-dependent dioxygenase], HEL, Helicase; POL, RNA dependent RNA polymerase; Hsp70h, Heat shock protein 70 homolog; CP, Coat protein; CPm, minor coat protein. Below the genome map the predicted sgRNAs are indicated by lines. ^a^Not present in Group VI variants. ^b^Highly divergent in Group VI and VI-like variants. ^c^Putative sgRNA.

Currently, the complete genomes of 10 distinct GLRaV-3 isolates representing four major groups of genetic variants are available (Table 2). All these genomes possess very long 5′UTRs of 510–802 nts and shorter, more conserved 3′UTRs; the features of these UTRs are further discussed in Section “Genetic Variants of GLRaV-3.” The consensus genome organization of the GLRaV-3 isolates from groups I-III encompassing 13 open reading frames (ORFs) is shown in Figure 4. The ORFs are designated 1a, 1b, and 2–12 according to the convention set out by Agranovsky et al. (1994). There is also a large, GC-rich intergenic region between ORFs 2 and 3 that is atypical of members of the family *Closteroviridae*. The genomes of isolates in variant group VI that have been characterized so far lack ORF2 (Bester et al., 2012a; Seah et al., 2012). Isolates from groups IV and V have yet to be fully sequenced.

**Table 2:** **Complete and near complete genomes of GLRaV-3**.

Isolate	GenBank accession #	Country	*Vitis vinifera* cultivar	Genome size (nt)	5′UTR	3′UTR	Group	Reference
NY-1	NC_004667	USA	Pinot Noir	17919*	158*	277	I	Ling et al. (2004)
621	GQ352631	South Africa	Cabernet Sauvignon	18498	737	277	I	Jooste et al. (2010)
WA-MR	GU983863	USA	Merlot	18498	737	277	I	Jarugula et al. (2010)
CL-766	EU344893	Chile	Merlot	17919*	158*	277	I	Engel et al. (2008)
GP18	EU259806	South Africa	Cabernet Sauvignon	18498	737	277	II	Maree et al. (2008)
623	GQ352632	South Africa	Ruby Cabernet	18498	737	277	II	Jooste et al. (2010)
PL-20	GQ352633	South Africa	Cabernet Sauvignon	18433	672	277	III	Jooste et al. (2010)
LN	JQ423939	China	Venus Seedless	18563	802	277	III	Fei et al. (2012)
CA7246	JQ796828	USA	Merlot	18552	737	274	VI	Seah et al. (2012)
GH11	JQ655295	South Africa	Cabernet	18671	737	264	VI	Bester et al. (2012a)
GH30	JQ655296	South Africa	Cabernet	18576	642	264	VI	Bester et al. (2012a)
139	JX266782	Australia	Sauvignon Blanc	18475	510	250	ND	Rast et al. (2012)

*Near complete genomes.

*ND, Not determined*.

The putative functions of the GLRaV-3 proteins encoded by ORFs 3–7 could be inferred by comparison to the homologous ORFs in the genomes of other positive-strand RNA viruses that contain a conserved “core” of replication genes and a “shell” of more variable genes encoding structural and accessory proteins (Dolja and Carrington, 1992). As is typical of the Alphavirus-like superfamily of viruses to which the family *Closteroviridae* belongs, the conserved core includes capping/methyltransferase, superfamily 1 RNA helicase, and RNA dependent RNA polymerase domains (Koonin and Dolja, 1993; Dolja et al., 2006) encoded by GLRaV-3 ORFs 1a and 1b (Ling et al., 2004). Indispensability of these ORFs for RNA replication was demonstrated using reverse genetics for two closteroviruses, LIYV (Klaassen et al., 1996) and BYV (Peremyslov et al., 1998). In addition, ORF1a of GLRaV-3 contains a papain-like leader protease (L-Pro) (Ling et al., 2004) that is implicated in RNA accumulation, virus invasiveness, and systemic spread of BYV (Peng and Dolja, 2000; Peng et al., 2003) and GLRaV-2 (Liu et al., 2009). Remarkably, GLRaV-3 ORF1a also harbors an AlkB domain (Maree et al., 2008) capable of RNA demethylation that is present in many RNA viruses infecting woody plants and proposed to repair viral RNA (Van den Born et al., 2008). Nevertheless, the functional role of different proteins encoded by GLRaV-3 can be studied using a biologically active, full-length cDNA clone that was recently reported (Jarugula et al., 2012).

There are no detectable homologs of the small protein putatively encoded by GLRaV-3 ORF2. The expression of this ORF is uncertain; as mentioned above, it is also missing in GLRaV-3 group VI isolates and seems unlikely to carry an essential function (Bester et al., 2012a; Seah et al., 2012). In contrast, the following five ORFs 3–7 comprise a quintuple gene module that is a conserved hallmark of the family *Closteroviridae* (Dolja et al., 2006). Of these, ORF3 codes for a small transmembrane protein for which the analogous protein of BYV is a cell-to-cell movement protein targeted to the endoplasmic reticulum (Peremyslov et al., 2004a).

As shown for several other closteroviruses, the ORF 4-encoded homolog of cellular HSP70 molecular chaperones (HSP70h) functions in cell-to-cell movement (Peremyslov et al., 1999) and assembly of the short virion tails typical of closteroviruses (Tian et al., 1999; Satyanarayana et al., 2000; Alzhanova et al., 2001; Peremyslov et al., 2004b). This protein is autonomously targeted to plasmodesmata in a myosin VIII-dependent manner (Avisar et al., 2008). The function of the ∼60 kDa protein encoded by ORF5 is similar to that of HSP70h; these two proteins likely cooperate in virion tail assembly and cell-to-cell movement (Alzhanova et al., 2007). The ORF6 encodes the *bona fide* CP that forms the long virion body, which is also required for cell-to-cell movement (Alzhanova et al., 2000). The last protein of this conserved quintet is a minor capsid protein (CPm) that is actually a main component of the virion tail (Agranovsky et al., 1995; Satyanarayana et al., 2004). Conspicuously, the C-terminal domain of ∼60 kDa protein, CP, and CPm all belong to a large family of proteins forming filamentous virions of plant viruses (Dolja et al., 1991; Boyko et al., 1992; Napuli et al., 2003). It is interesting to note that the order of the CP- and the CPm-encoding ORFs in GLRaV-3 is the same as in the bi-partite criniviruses (e.g., LIYV) but reversed compared to viruses in the genus *Closterovirus* (e.g., BYV and CTV) (Karasev, 2000). Although these proteins have not been completely characterized for GLRaV-3 it is clear that the functions of the HSP70h, ∼60 kDa protein, and CPm in the virion tail assembly and cell-to-cell movement of closteroviruses are genetically inseparable, and the tail assembly can be conceptualized as a closterovirus-specific movement device (Dolja, 2003; Peremyslov et al., 2004b).

The functions of the remaining ORFs 8–12 could not be inferred by sequence analysis because their products are not conserved outside the genus *Ampelovirus* (Ling et al., 1998). However, by analogy to similarly located ORFs of other members of the family *Closteroviridae*, GLRaV-3 ORF 8, 9, and 10-encoded proteins could be involved in suppression of the host RNA interference defense (Reed et al., 2003; Lu et al., 2004; Chiba et al., 2006) and viral long-distance transport (Prokhnevsky et al., 2002). The recent work by Gouveia et al. (2012) provided experimental support for the suppressor activity of the ORF10 product p19.7 (p20B) in *N. benthamiana*. This protein was also proposed to be a viral pathogenicity determinant (Gouveia and Nolasco, 2012), an activity rather typical of viral suppressors of RNA interference (Voinnet, 2005). The small ORFs 11 and 12 are unique to GLRaV-3 and are not present in other members of the family *Closteroviridae*. Because these ORFs are very diverse among GLRaV-3 variant groups, they are unlikely to specify conserved functions. The functional characterization of ORFs 8–12 and the AlkB domain is a major challenge for future research. The recent development of a biologically active, full-length cDNA clone will aid in determining the functions of these GLRaV-3 proteins (Jarugula et al., 2012).

## GLRaV-3 Genome Expression and Replication

The replication-associated proteins of GLRaV-3 encoded by ORFs 1a and 1b are translated directly from the capped genomic RNA, analogously to BYV (Karasev et al., 1989). Translation of the ORF 1b-encoded RdRp likely involves a translational +1 frameshift (Agranovsky et al., 1994; Ling et al., 2004). The resulting products of ORF 1a and ORF 1a + 1b translation are likely processed by a papain-like L-Pro; this processing is critical for the RNA replication in BYV and GLRaV-2 (Peng and Dolja, 2000; Liu et al., 2009). Interestingly, the BYV L-Pro co-localizes with the vesicular network derived from the endoplasmic reticulum (Zinovkin et al., 2003), which, similar to other positive-strand RNA viruses, is recruited by replicase proteins to form viral RNA replication complexes (Den Boon and Ahlquist, 2010). Although not confirmed experimentally, the GLRaV-3 RNA replication likely occurs via recognition of the structural promoter elements formed by the 3′- and 5′UTRs present in the positive and negative strands of the viral RNA as was shown for CTV (Satyanarayana et al., 2002; Gowda et al., 2003).

Similar to other characterized members of the family *Closteroviridae*, the GLRaV-3 ORFs localized downstream of the ORF 1b are expressed via formation of a nested set of sgRNAs that are 3′-colinear with the gRNA (Jarugula et al., 2010; Maree et al., 2010). Each of these sgRNAs serves as a monocistronic messenger for translation of the corresponding 5′-proximal ORF. The sgRNAs are likely transcribed from a genomic RNA by the viral replicase that recognizes internal sgRNA promoters (Miller and Koev, 2000), although the exact mechanism of this process seems to be more complicated in closteroviruses than previously anticipated (Ayllón et al., 2004). Early studies of GLRaV-3 infection suggested the production of multiple sgRNAs (Hu et al., 1990; Rezaian et al., 1991; Saldarelli et al., 1994; Ling et al., 1997), but only recently have these RNAs been characterized in some detail for two different isolates (Jarugula et al., 2010; Maree et al., 2010). In particular, a Northern blot analysis of dsRNA was used to demonstrate the 3′-co-terminal structure of the three sgRNAs corresponding to ORFs 4, 5, and 6 (Maree et al., 2010). The study by Jarugula et al. (2010) showed that sgRNAs expressing ORF6 (CP), ORF8 (p21), ORF9 (p20A), and ORF10 (p20B) are the most abundant viral RNAs present in a GLRaV-3-infected grapevine (*V. vinifera* cv. Merlot). Among these, the sgRNA corresponding to ORF10 (p20B) accumulated to the highest level, followed by sgRNAs encoding products of the ORF8 (p21), ORF9 (p20A), and ORF6 (CP). These results suggest that temporal and quantitative regulation of GLRaV-3 sgRNA transcription occurs during the virus infection cycle, leading to differential expression, and/or accumulation of sgRNAs in a distinct regulation pattern.

The 5′-transcriptional start sites (TSS) for several GLRaV-3 sgRNAs were determined for isolates GP18 and WA-MR that belong to two different genetic variant groups (Jarugula et al., 2010; Maree et al., 2010). Although the techniques used were different between the two studies (RLM-RACE and 5′RACE respectively), identical results were obtained with the exception of the ORF9 sgRNA where start sites differed by one nucleotide (Table 3). All the mapped 5′-terminal nucleotides were purines and were conserved between the two isolates. The 5′UTRs of the characterized sgRNAs were variable in size with no detectable conserved sequences surrounding the TSS (Jarugula et al., 2010; Maree et al., 2010). This is in contrast to CTV and BYV, where conserved secondary structure elements were proposed to occur in the sgRNA promoters (Peremyslov and Dolja, 2002; Ayllón et al., 2004; Vitushkina et al., 2007). There also appears to be no correlation between the length of 5′UTR and the accumulation levels of the GLRaV-3 sgRNAs, suggesting that transcriptional regulation of the genus *Ampelovirus* is likely distinct from that of the genus *Closterovirus* (Jarugula et al., 2010).

**Table 3:** **Position of transcription start sites of GLRaV-3 sgRNAs**.

ORF	ATG	Maree et al. (2010)		Jarugula et al. (2010)	
		Transcription start site^a^	Predicted sgRNA	Transcription start site^b^	Predicted sgRNA
2	9287				
3	10509				
4	10665	G-10477	sgRNA(ORF3/4)		sgRNA (HSP70h)*
5	12307	G-12185	sgRNA(ORF5)		sgRNA (p55)*
6	13848	A-13800	sgRNA(ORF6)	A-13800	sgRNA (CP)
7	14852	G-14815	sgRNA(ORF7)		sgRNA (CPm)*
8	16296	A-16273	sgRNA(ORF8)	A-16273	sgRNA (p21)
9	16850	G-16754	sgRNA(ORF9)	A-16755	sgRNA (p20A)
10	17390	A-17265	sgRNA(ORF10-12)	A-17265	sgRNA (p20B)
11	17932				sgRNA (p4)
12	18039				sgRNA (p5)

*^a^5′ end determined by RLM-RACE (Ambion) and mapped on the genome of isolate GP18 (GenBank: EU259806)*.

*^b^5′ end determined by 5′ RACE (Invitrogen) and mapped on the genome of isolate WA-MR (GenBank: GU983863)*.

*Putative sgRNAs.

## Genetic Variants of GLRaV-3

The genetic variability of GLRaV-3 has been studied extensively in recent years and research worldwide showed the existence of several genetic variants of GLRaV-3. Earlier studies on the genetic variability used single-stranded conformation polymorphism (SSCP) combined with sequence analysis of different genomic regions (Jooste and Goszczynski, 2005; Turturo et al., 2005). Turturo et al. (2005) investigated the genetic variability of three genomic regions; RdRp, HSP70h, and CP genes, for 45 GLRaV-3 isolates from 14 different countries. Their results for the RdRp and HSP70h regions showed that 10% of the isolates analyzed, had mixed variant infections, whilst 15% of the isolates had mixed infections when the CP region was analyzed (Turturo et al., 2005). Multiple alignment of sequences deposited in GenBank revealed that the sequences used in the Turturo study had nucleotide identities of above 90% between isolates in the regions studied. Using SSCP analysis, Jooste and Goszczynski (2005) classified two divergent GLRaV-3 variant groups, I and II, represented by isolates 621 and 623.

Sequence comparisons between isolates using different genome regions confirmed the genetic variation shown by earlier studies and indicated a greater diversity than originally estimated. Diversity studies using a portion of the RdRp or the HSP70h revealed isolates clustering into three groups. These groups had a higher than 95% similarity, between 90 and 95% similarity or approximately 70% similarity at the nucleotide level (Angelini et al., 2006; Soule et al., 2006; Prosser et al., 2007; Engel et al., 2008). Sequence data from a survey of GLD-associated viruses, using the HSP70h, showed a range of identity between 74.1 and 100% at the nucleotide level and 85.9–100% at the amino acid level between GLRaV-3 isolates from different geographic regions (Fuchs et al., 2009). Phylogenetic analysis of the HSP70h gene showed at least five possible variant groups (Fuchs et al., 2009). Subsequent studies using phylogenetic analysis of various genome regions, predominantly the CP but also HSP70h, CPm, p55, and RdRp, also confirmed five variant groups as well as identified diverse isolates currently grouped in group VI and more distantly related isolates suggesting a group VII (Chooi et al., 2009, 2013a; Gouveia et al., 2011; Sharma et al., 2011; Wang et al., 2011; Bester et al., 2012a; Seah et al., 2012). Due to limited sequence information and for the purpose of this review all isolates related to group VI, but divergent, will be referred to as group VI-like. Genetic variant groups I, II, and III were shown to be consistent between these studies but direct comparison of groups IV and V was not possible since these studies did not use the same genome regions or isolate sequences. Full-length genome comparisons of isolates from different variant groups indicated significant sequence variation in some genomic regions compared to others, as well as length variation in the 5′UTR, highlighting the risk of phylogenetic analysis using partial genome sequences. Although the biological relevance of the current genetic variant group classifications, based on partial sequences, remains to be determined, it will be beneficial for future studies to use a unified system to be able to draw direct comparisons between studies.

### Distribution of GLRaV-3 variants in GLD affected vineyards

Several studies investigated the distribution of specific GLRaV-3 variants in vineyards. The distribution can be influenced by many factors such as specific virus-vector interactions, prevailing wind direction, combinations of GLRaV-3 variants, use of virus-infected planting material, and viticultural practices. In a South African study, group II variants occurred predominantly in the vineyards surveyed; suggesting that group II variants are most widespread (Jooste et al., 2011). In the same study, the natural spread of GLRaV-3 variants along the rows of a vineyard as well as the distribution patterns was documented (Jooste et al., 2011). Recently, group VI variants were identified in South African vineyards (Bester et al., 2012a) and their prevalence in some regions was shown (Jooste et al., 2012). Group I genetic variants were found to be dominant in a Chinese survey (Farooq et al., 2012), while in Portugal groups I and II were the most common (Gouveia et al., 2009). In a New Zealand study, group I and VI-like (similar to NZ2) variants occurred predominantly in a germplasm and commercial vineyard block, while the group VI variant was only found in high numbers in the germplasm block (Chooi et al., 2013b).

In a study of Napa Valley vineyards (Sharma et al., 2011), 27% of the GLRaV-3 isolates characterized were group I variants, while 13 and 31% were group II and III variants, respectively. The study reported that mixed variant infections occurred in 21% of infected samples and that single variant infections with group I and III were the most prevalent (Sharma et al., 2011). The transmission dynamics of variants I and VI in Napa Valley was tested (Blaisdell et al., 2012). The study found that vector transmission of the group VI variant alone was more frequent, followed by transmission with mixed infections of the two, while transmission with the group I variant alone was the least common. It should be highlighted that this is the first evidence that GLRaV-3 variants are biologically distinct. We expect that future work will be able to identify biological differences among the various variants within this species, if they exist.

### Genomic variability between GLRaV-3 isolates

**Figure 5:**
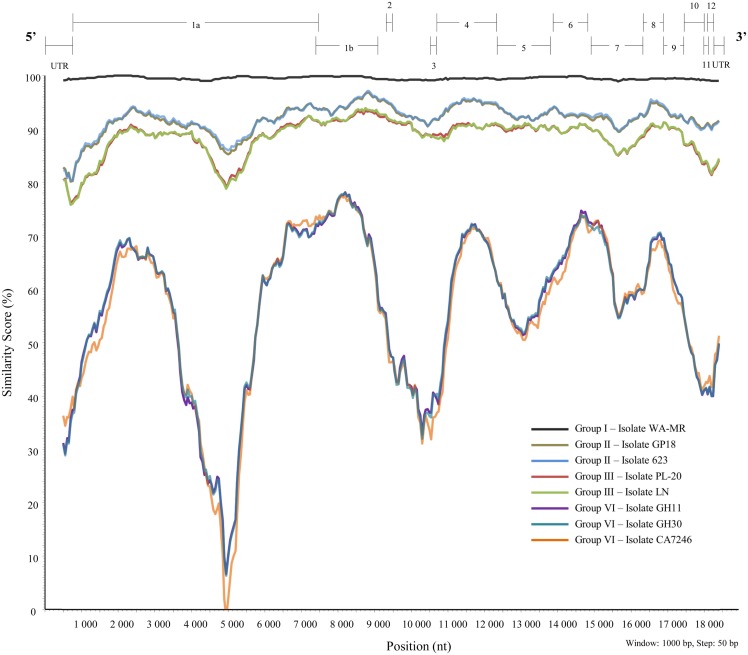
**Similarity plot constructed from a multiple alignment of nine full-length sequences representing six well-defined variant groups of GLRaV-3 using SimPlot, PHYLIP (Pylogeny Inference Package) v3.5.1 (Lole et al., 1999)**.

A similarity plot (Figure 5) was constructed using isolate 621 from group I as reference sequence with a multiple sequence alignment constructed with BioEdit 7.0.5.3 (Hall, 1999) of full genome sequences of representatives of the different variant groups (I, II, III, and VI). Currently, there are no full-length sequences for representatives of groups IV and V. Genomic regions with high variability, or major variation between variant groups are discussed below.

#### 5′UTR

Variability in the 5′UTR was first described between isolates of groups I, II, and III after full-length genome sequences were generated for four South African GLRaV-3 isolates (Maree et al., 2008; Jooste et al., 2010). Isolate 621, representing group I, and isolates GP18 and 623, representing group II, all had a 5′UTR of 737 nt. The third variant, represented by isolate PL-20, contained a shorter 5′UTR of 672 nt, resulting in a genome that is 65 nt shorter than the sequences of group I and II variants (Jooste et al., 2010). The WA-MR sequence from Washington showed a similar 5′UTR sequence and length of 737 nt (Jarugula et al., 2010) to that of the other group I isolate 621. Another full-length sequence of a representative of group III, isolate LN (GenBank: JQ423939), contained a much longer 5′UTR compared to PL-20. The recent full-length sequences of group VI variants from South Africa (Isolates: GH11 and GH30), and the USA (Isolate: CA7246) revealed 5′UTRs of 737 nt for isolates GH11 and CA7246, and a shorter length 5′UTR for isolate GH30 (Bester et al., 2012a; Seah et al., 2012). To determine if there are sequence or structural conservation within the 5′UTR it will be important to also have sequence information from groups IV and V.

#### ORF2

Sequence data showed no ORF homologous to the GLRaV-3 ORF2 in isolates from variant group VI. This was verified in sequence data of isolates GH11 and GH30 from South Africa (Bester et al., 2012a), partial sequence of NZ-1 from New Zealand and the Californian isolate CA7246 (Seah et al., 2012). The function of ORF2 in variants I–V remains unknown.

#### ORF11 and 12

The position and size of ORF11 is common to all GLRaV-3 variants from groups I–IV and VI. However, GLRaV-3 variants from group IV require an alternative start codon (ACG) (Wang et al., 2011). Moreover, based on sequence alignments, group VI isolates have frameshifts within ORF11 when compared to other GLRaV-3 variants from groups I–IV. This leads to changes in the amino acid sequence from amino acid 5 onward (Bester et al., 2012a; Chooi et al., 2013a). For the NZ2 isolate, the ORF11 is 18 nt longer than groups I–IV and VI resulting in polypeptide that is six amino acids longer. Compared to other GLRaV-3 variants, translation of the NZ2 ORF11 would occur in the same frame, however translation is predicted to start 3 nt upstream (1 nt overlap of ORF10) and terminate 15 nt downstream (14 nt overlap of ORF12) from the predicted start and stop sites of other GLRaV-3 variants (Chooi et al., 2013a).

The predicted start position of ORF12 is common to all known GLRaV-3 variants. However, a frameshift within the ORF12 of group VI variants and isolate NZ2 leads to a premature stop codon and in turn a reduction in size (Bester et al., 2012a; Chooi et al., 2013a). The ORF12 of GLRaV-3 variants from groups I to IV is 183 nt in size, which corresponds to a 61 amino acid polypeptide. In contrast, the ORF12 of group VI variants and isolate NZ2 is 18 nt and 12 nt shorter than groups I–IV, and as a result produces smaller 55 and 57 amino acid polypeptides, respectively.

Sequence variation along the GLRaV-3 genomic RNA is unevenly distributed. In particular, high sequence variation is evident for ORFs 11 and 12. Nineteen complete ORF11 sequences from isolates representative of groups I–IV and VI, and the corresponding trimmed sequence for the group VI-like NZ2 isolate were compared. High ORF11 amino acid variation between phylogenetic groups was also observed, as the average amino acid inter-group variations for groups I–IV ranged between 14.6 and 38.0% and group VI showed 68.1–74.2% divergence when compared to groups I–IV isolates. Isolate NZ2 showed an average 81.9 and 65.3% amino acid divergence compared to isolates from group III and group VI, respectively, while 86.1% compared to groups I, II, and IV isolates. This particularly high genetic variation observed in ORF11 supports the premise that this ORF is under neutral evolution (Wang et al., 2011), and that the predicted ORF is either not translated or is non-essential for virus infection (Seah et al., 2012).

In contrast to ORF11, less sequence variation was observed for ORF12. The average nucleotide inter-group variation for groups I–IV ranged between 6.0 and 17.4%, while isolates from group VI and group VI-like (Isolate NZ2) showed an average of 34.5–38.7% inter-group variation when compared to groups I–IV isolates. The average variation between isolates of groups VI and VI-like (Isolate NZ2) was 28.3%. It is evident that, like ORF2, genetic diversity studies indicate that functional research needs to be performed to better understand the role, if any, of ORF11 and 12 in GLRaV-3 biology.

#### 3′UTR

The GLRaV-3 3′UTR length varies; group I–III isolates are 277 nt, except for isolate 139 which is 250 nt; while the 3′UTR of isolates from group VI are 264 nt, except for CA7246 which is 274 nt. The 3′UTR of isolate NZ2 (group VI-like) is 289 nt in length (Chooi et al., 2013a). Based on the 277 nt 3′UTR sequence, the average nucleotide identity between isolates from groups I–III is 96.4%, while isolate NZ2 (group VI-like) only shares on average 78.7% nucleotide identity to isolates from groups I–III. Based on the shorter 264 nt group VI 3′UTR sequence, group VI isolates have on average 20.8 and 12.3% nucleotide variation to isolates from groups I–III, and the group VI-like (Isolate NZ2) respectively. The overall average nucleotide identity between all isolates from phylogenetic groups I–III, VI, and VI-like is 88.7%. The observed variability in the 3′UTR length and nucleotide identity is similar to BYV, where the 3′UTR of isolates U and Cal are 166 and 182 nt, respectively, and share 89.6% nucleotide identity (Agranovsky et al., 1994; Peremyslov et al., 1998).

Potential *cis*-acting elements that are critical for virus replication have likely conserved primary sequence and/or secondary structures, similar to conserved replication signals found in the 3′UTR of CTV. Sequence variation along the GLRaV-3 3′UTR is unevenly distributed. The highest sequence variation occurs at the 5′ end of the 3′UTR (region closest to ORF12) as nucleotide identities between isolates from phylogenetic groups I to III, VI and VI-like (Isolate NZ2) decrease to as low as 50%. While two regions from nucleotides 18,320–18,382, and 18,430–18,498 (based on GP18 numbering) have low sequence variation, as the average nucleotide identities between group I–III, VI, and VI-like (Isolate NZ2) isolates increase to 90% or greater. Thus, these areas of high conservation may represent possible cis-acting elements important for controlling GLRaV-3 replication.

### Recommendation for naming and distinguishing variant groups of GLRaV-3

**Figure 6:**
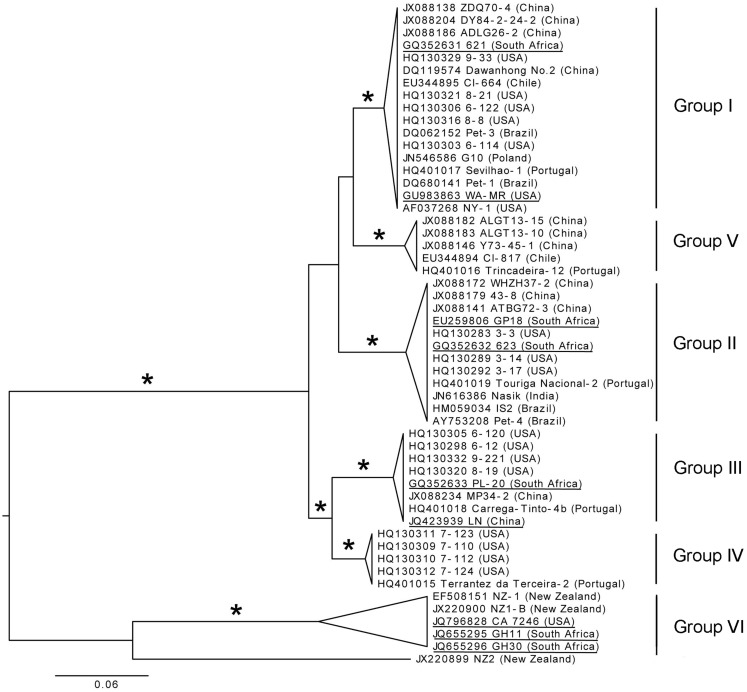
**Phylogenetic tree of full-length CP gene from representative GLRaV-3 isolates (Isolate NZ1, GenBank: EF508151, is a partial sequence)**. Proposed GLRaV-3 variant groups are shown with roman numerals. Maximum likelihood tree is shown, but analyses with distance and maximum parsimony methods provide similar topology. The tree is midpoint rooted for presentation and asterisks indicate ≥75% branch support with all three methods. Accession number, isolate name, and country where samples were collected are shown; fully sequenced genomes are underlined for reference. The phylogenetic analysis was performed with PAUP* (Swofford, 2003) and image generated with FigTree (Rambaut, 2006).

The diversity in the CP gene was examined based on the assumption that viral CPs evolved more rapidly than proteins involved in replication and expression of virus genomes, providing better phylogenetic resolution (Callaway et al., 2001). A total of 196 full-length CP sequences from Brazil, China, Chile, India, New Zealand, Poland, Portugal, South Africa, and the USA that are deposited in GenBank were aligned. From this alignment, sequences representing different genetic groups of GLRaV-3 were arbitrarily selected to construct a phylogenetic tree (Figure 6). Six well-supported phylogenetic groups were detected in the analysis of full-length CP gene sequences of 53 isolates. GLRaV-3 variant groups I–VI were confirmed as previously identified along with group VI-like isolates that might be classified into new variant groups when more supporting data is available.

Nucleotide sequences of the CP region were analyzed and high homology, with variation of less than 2.2% within variant groups I–V, were found. Nine partial CP nucleotide sequences (Isolates: 7-1006, 7-1010, 21-9, 22-2, 43-12, 43-15, 44-2, 22-15, 21-12) from the Sharma et al. (2011) study were compared to other isolates in variant group VI. All these isolates were similar to the Californian isolate, CA7246, except isolate 43-15 (GenBank: JF421951). The Californian isolates (excluding 43-15) have 99.9–100% homology. The CP sequences of isolates 43-15 (partial), CB19 (partial, GenBank: EF445655) and 139 (GenBank: JX266782), grouped together most related to group VI but separate from isolates with the same geographical origin (Group VI-like). The New Zealand isolates, NZ-1 and NZ1-B, and South African isolates, GH11 and GH30, showed nucleotide divergence of 7.1% and 7.9–8.3% to the Californian isolates, respectively. The New Zealand and South African isolates from group VI differed by 7.1%. This illustrates that within group VI, genetic variation is greater than for the other variant groups, especially when partial nucleotide sequences from the group VI-like isolates CB19, 43-15, and 139 are included. The newly described isolate NZ2 showed a 17.7–18.7% nucleotide divergence to the group VI isolates.

It is clear from Figure 6 that two main phylogenetic clades exist. Firstly, the clade that include isolates from groups I–V, and secondly, the clade containing the group VI and group VI-like isolates The classification of the GLRaV-3 phylogenetic groupings should be reevaluated when more sequence data are available, considering the significantly higher genetic variability within group VI clade compared to the other groups (Chooi et al., 2013a).

The availability of sequence data is crucial to classify GLRaV-3 variant groups. Analysis of the available data suggests that there are at least six well-supported phylogenetic groups within GLRaV-3 populations worldwide (Figure 6). We propose that these phylogenetic groups be named using a Roman numeral classification system, i.e., groups I–VI, to provide harmonization. In two studies (Sharma et al., 2011; Wang et al., 2011) letters were used for naming groups with groups 3a, 3b, 3c, 3d, 3e, and 3g being synonyms to groups I, II, III, IV, VI, and V, respectively while other studies have also used isolate names to designate variant groups. We further propose that full-length sequences, underlined in Figure 6, be included for each genetic group in future phylogenetic studies.

We suggest that ascending, consecutive Roman numerals be used for maintenance of shared terminology by the community. Based on partial sequences and the number of new variants recently found throughout the world, we expect that more genetic variation will be revealed in the future and that more GLRaV-3 phylogenetic groups will be identified.

## Detection

The identification of disease-associated viruses has proven to be challenging since most diseased grapevines are infected with more than one virus. This is further complicated as unrelated viruses can cause similar disease symptoms, new infections typically have a low virus titer, viruses are often unevenly distributed in infected vines, and symptoms in some white cultivars and rootstocks are less noticeable. To date several techniques have been applied to detect viruses associated with GLD in plant material, including biological indexing, serology, nucleic acid-based methods, and next-generation sequencing.

### Biological indexing

Until the late 1980s, the only reliable method of testing for GLD was hardwood indexing on biological indicators. A small chip bud from the selection to be tested (the candidate vine) is grafted to an indicator grapevine cultivar (Rowhani and Golino, 1995; Constable et al., 2010). The indicator plants with the newly grafted material are planted in the field and observed for at least two seasons for the development of virus disease symptoms (Weber et al., 2002). *V. vinifera* cvs Cabernet Franc, Pinot noir, Cabernet Sauvignon or Barbera may be used as indicator host, depending upon personal preferences and/or climatic conditions under which the indicator is grown. Green-grafting is another biological indexing technique used to screen grapevine material for viruses including GLRaV-3 (Taylor et al., 1966; Walker and Golino, 1999; Pathirana and McKenzie, 2005). Green scions or buds are grafted onto green shoots and has a higher success rate and is capable of overcoming the graft incompatibility sometimes experienced between distantly related *Vitis* species (Walker and Golino, 1999; Walter et al., 2008). Biological indexing onto woody indicators is labor intensive, time-consuming, and dependent on the successful inoculation of associated viruses (Weber et al., 2002). Uneven distribution of the virus, strain variation within the associated virus species, low virus titer, and the lack of symptom expression can also affect the results obtained with indexing (Rowhani et al., 1997; Constable et al., 2010). Biological indexing detects the disease rather than the associated viruses and although this technique can be a successful detection method, it requires a skilled virologist for disease confirmation and sometimes relies on subjective visual observations.

### Serology

Many different formats of serological diagnostic techniques have been developed; these include enzyme-linked immunosorbent assay (ELISA), immunofluorescence (IF), and immuno-strip tests (Schaad et al., 2003). For a historical overview of GLD related antisera and monoclonal antibodies, see Gugerli (2009). Although, ELISA is not as sensitive as nucleic acid-based techniques its robustness and scalability makes it popular for routine testing by industry for the detection of GLD associated viruses in grapevines used for propagation. It is a robust, simple, and cost-effective detection method that is scalable for high-throughput processing (O’Donnell, 1999; Ward et al., 2004). Disadvantages of the technique are that it has a high developmental cost and is not as sensitive as nucleic acid-based methods (O’Donnell, 1999). Since the first antiserum was produced against closterovirus-like particles (Gugerli et al., 1984) several groups have produced their own polyclonal antisera or monoclonal antibodies to develop ELISAs to detect GLRaV-3 specifically (Teliz, 1987; Zee et al., 1987; Gugerli et al., 1990; Goszczynski et al., 1995; Ling et al., 2000, 2001). Currently, it is unknown if all industry recommended ELISA kits can detect all the newly reported genetic variants. The robustness of ELISA makes it likely that all genetic variants can be detected. In South Africa, the industry standard kit (locally produced) could detect GLRaV-3 from genetic variant groups I, II, III, and VI with equal efficiency (Bester, 2012). However, in New Zealand it was found that genetic variants from group VI and those related to isolate NZ2 were weakly detectable and required modifications of protocols (Cohen et al., 2012).

### Nucleic acid-based methods

Nucleic acid-based methods have increasingly been used in recent years to develop diagnostic assays for plant pathogens. Reverse transcription-PCR (RT-PCR) was developed for pathogens with RNA genomes (Ward et al., 2004) such as most of the known viruses in grapevine, including GLRaV-3. The genomic RNA of GLRaV-3 is found to be heterogeneous and up to date six genetic variants of the virus have been reported (Jooste et al., 2010; Gouveia et al., 2011; Wang et al., 2011; Kumar et al., 2012). Due to this genomic variability, two different multiplex PCRs were described for the detection and differentiation of four and five of the genetic variant groups of GLRaV-3, respectively (Bester et al., 2012b; Chooi et al., 2012). Another approach to PCR is called immunocapture PCR (IC-PCR). It is used for the detection of GLRaV-3 by utilizing antibodies, produced against the recombinant major CP, to immobilize the virus on the surface of a microfuge tube and continue with RT-PCR amplification (Nolasco et al., 1997; Ward et al., 2004; Engel et al., 2008). Spot-PCR has also been successfully applied for the detection of pathogens in woody plants, where a small drop of unbuffered sap from grapevine leaf petioles is placed on filter paper and used as the template for PCR (La Notte et al., 1997; Dovas and Katis, 2003; Osman and Rowhani, 2006). Another alternative to conventional PCR is the Loop-mediated amplification of nucleic acid (LAMP) technique. The LAMP method relies on the isothermal amplification of a target sequence by a strand displacing DNA polymerase and four primers with six target areas. This method has been applied for the detection of viruses including GLRaV-3 by adding reverse transcriptase to the LAMP protocol (RT-LAMP) (Nolasco, 2010; Pietersen and Walsh, 2012).

The quantification of target DNA has been simplified with the introduction of real-time PCR where unknown samples are quantified absolutely or relatively by comparing it to a standard DNA sample or to a reference gene (Feng et al., 2008). Different fluorescent probe-based chemistries have been developed of which TaqMan probes are more commonly used for grapevine virus detection. A real-time TaqMan RT-PCR assay was developed for the simultaneous detection of GLRaV-1, -2, -3, and -4 and some of the related GLRaV-4 strains and shown to be more sensitive than conventional one-step RT-PCR (Osman et al., 2007). TaqMan low-density arrays have also been introduced as a modified method of real-time TaqMan PCR. This method uses microtiter plates with dried TaqMan PCR primers/probes complexes added to the wells. It was developed for the detection of 13 different grapevine viruses (Osman et al., 2008). Recently, real-time RT-PCR high-resolution melting (HRM) curve analysis has been applied to detect and differentiate the genetic variant groups of GLRaV-3 utilizing the DNA binding dye, SYTO 9, as an alternative to TaqMan probes (Bester et al., 2012b). Other methods used to differentiate between genetic variants of GLRaV-3 include single-strand conformation polymorphism (SSCP) profiles and asymmetric PCR-ELISA (APET) (Jooste and Goszczynski, 2005; Turturo et al., 2005; Gouveia et al., 2009, 2011; Jooste et al., 2010).

More recently, oligonucleotide microarray analysis has been developed and used to detect several viruses or genes at the same time. A grapevine microarray, containing 570 unique probes designed against highly conserved and species-specific regions of 44 plant viral genomes could accurately detect 10 grapevine viruses (Engel et al., 2010). Three members of the family *Closteroviridae*, e.g., GLRaV-4, -7, and -9 were detected for the first time in Chilean grapevines using this microarray (Engel et al., 2010). This approach provides a powerful tool for high-throughput screening that can be useful for plant certification purposes. As more viral sequences become available, additional probes can be designed, raising the possibility of detecting divergent virus isolates. However, microarray technologies in general are still expensive and require extensive data analysis. Recently, the successful application of macro-array methodology was demonstrated as an alternative to microarray technology. Thompson et al. (2012) provided an unbiased multiplex detection system using a single robust macro-array platform for grapevine viruses. The relative simplicity and robustness of this methodology will be accessible to most molecular biology laboratories due to the only major equipment required being a thermocycler and a hybridization oven. This platform can differ in detection sensitivity in comparison to RT-PCR, but can complement other molecular detection methods by providing a multiplexing component (Thompson et al., 2012).

### Next-generation sequencing

Present grapevine disease diagnostics rely on ELISA or nucleic acid-based methods to target viruses that have in the past been associated with diseases (Adams et al., 2009). Although these techniques can be very specific and reliable, they do not take into account the contribution of other known or unknown viruses that may be involved in the disease etiology. Different virus variants can also exist that may go undetected if highly specific RT-PCR protocols are used. The use of metagenomic sequencing to establish the total viral complement of a sample has been shown to avoid these limitations of current plant virus diagnostics (Adams et al., 2009; Al Rwahnih et al., 2009; Kreuze et al., 2009; Coetzee et al., 2010). Second generation or next generation sequencing (NGS) instruments have been developed, avoiding the limitations associated with Sanger sequencing (Hall, 2007; Mardis, 2008). The use of universal adaptors, rather than sequence specific primers, makes NGS specifically suitable to sequence all the genetic material present in a sample without prior knowledge of the organisms present (Hall, 2007; Mardis, 2008; Tucker et al., 2009). Although NGS is currently not used for GLRaV-3 diagnostics, two studies have applied NGS successfully to identify known and novel viruses from diseased plant material. Coetzee et al. (2010) established the viral profile of a severely diseased vineyard and identified a new GLRaV-3 variant that was not previously detected in South Africa. A Canadian research group also used Illumina NGS reads to assemble a complete genome sequence of GLRaV-3 (GenBank: JX559645). These studies indicate the usefulness of NGS technologies as a diagnostic tool to identify a plant virus when no prior knowledge of the virus is available. Next-generation sequencing is still relatively expensive to be used for routine diagnostics. However, data generated can be used to develop more accurate diagnostic assays since NGS can provide information regarding disease complexes, dominant variants of viral species and an indication of the frequency of viruses found in infected material.

## Host-Pathogen Interactions

### Transmission of GLRaV-3

The vector transmission biology of GLRaV-3 has been poorly characterized despite its obvious importance to disease spread under natural conditions. Spread of GLRaV-3 through contaminated plant material is still widespread and of significant economic and quarantine importance. Strategies to limit such virus dissemination are based on the production of clean propagative material through certification programs and educational efforts promoting the planting of certified accessions (Rowhani et al., 2005). In addition, vine-to-vine transmission of leafroll via dodder (*Cuscutacampestris*) is also possible for experimental purposes (Woodham and Krake, 1983). There is no evidence of GLRaV-3 mechanical transmission through pruning or other plant management practices. Here we focus on the vector transmission of GLRaV-3, which is expected to be the only means of pathogen spread after establishment of a new healthy vineyard. A review on the biology of grape-colonizing mealybugs is available elsewhere (Daane et al., 2012).

Work on the vector transmission of GLRaV-3 was initiated by Rosciglione and Gugerli (1987) and Engelbrecht and Kasdorf (1990), who demonstrated that the vine mealybug (Hemiptera, Pseudococcidae), *Planococcus ficus*, was a vector of GLD. This work had two important impacts on the academic and viticulture communities; it promoted new studies that led to the identification of several new insect vectors of GLRaV-3 and further work on disease spread in the field (reviewed by Charles et al., 2006b). Transmission of GLRaV-3 has been demonstrated for various species of mealybugs (Pseudococcidae) and a few species of soft scale insects (Coccidae) (list of experimental vectors can be found in Tsai et al., 2010), but little is known about parameters that affect the transmission efficiency of this virus. Although soft scale insects are experimental vectors of GLRaV-3, they are not considered to be epidemiologically important and are not discussed in detail here. Despite the limited amount of work characterizing GLRaV-3 transmission by mealybugs, important insights have been gained through experimental research. It appears that first instar nymphs are more efficient vectors of GLRaV-3 than older nymphs or adults (Petersen and Charles, 1997; Tsai et al., 2008). These finding may be influenced by the difficulty associated with handling adult mealybugs. The removal of adults from feeding sites may result in breakage of their long stylets that are still inserted into plants rendering them unable to feed. On the other hand, differences in probing behavior between adults and nymphs may also explain these observations (Cid and Fereres, 2010; Sandanayaka et al., 2012). Regardless, because adults are largely immobile and small nymphs may be easily dispersed, including via wind (Barrass et al., 1994), the young life stages are expected to be responsible for disease spread in the field. The role of adult mealybugs in disease spread is not well understood but the subterranean survival of viruliferous mealybugs on root remnants has significant implications for disease management especially where vineyards are replanted (Bell et al., 2009). Because mealybugs have feeding tissue preferences that vary based on species and season, efforts have been made to compare the transmission efficiency of insect vectors feeding on different plant tissues. However, no effect was found when insects were confined on different tissues for virus acquisition and inoculation (Tsai et al., 2011).

Insect-borne plant viruses have a myriad of interactions with their respective vectors (Nault, 1997). There is no knowledge on the molecular interactions between GLRaV-3 and any of its vectors. However, temporal aspects of transmission such as the time required for virus acquisition or inoculation, as well as retention, allow general inferences on the mode of pathogen transmission. Cabaleiro and Segura (1997) tested the effect of time on mealybug virus acquisition and inoculation, with acquisition only occurring after 3 days of plant access, while inoculation by mealybugs reared on infected plants did not occur after 24 h. The loss of infectivity after 24 h is representative of a non-persistently or semi-persistently transmitted virus, although a 3-day minimum acquisition access period is not. Further studies include the detection of GLRaV-3 using IC-RT-PCR of dissected organs of *P. citri* and immunogold labeling and transmission electron microscopy to identify the location of the virus in the primary salivary glands (Cid et al., 2007). On the other hand, Douglas and Krüger (2008) reported that 1 h and 30 min were enough for acquisition of GLRaV-3 by *P. longispinus*. More recent work with the same vector and virus species suggested that 24 h were necessary for pathogen acquisition (Sandanayaka et al., 2012). Such contrast in results is not unexpected for poorly studied systems with low transmission rates that are difficult to manipulate experimentally, primarily due to small sample sizes. Furthermore, differences in experimental conditions may explain some of these discrepancies. The small amount of work on the transmission biology of GLRaV-3 represents a significant gap in knowledge.

The first study aimed at addressing several temporal aspects of GLRaV-3 transmission simultaneously used *P. ficus* as an experimental vector (Tsai et al., 2008). In that study transmission efficiency peaked with acquisition and inoculation access periods of 24 h. In addition, the virus was retained and transmitted by insects up to 4 days after acquisition; molting; and/or loss of virus over time may have resulted in loss of infectivity. These are characteristic hallmarks of semi-persistently transmitted viruses, where transmission efficiency increases with hours of plant access period, and viruses are retained in vectors over a limited number of hours or days (Ng and Falk, 2006). For *Lettuce infectious yellows virus*, another member of the family *Closteroviridae*, the cibarium of its whitefly vector was identified as the likely virus retention site (Chen et al., 2011). The foregut of mealybug vectors is expected to be the retention site for GLRaV-3, but semi-persistently transmitted viruses may also bind to the tip of stylets (Uzest et al., 2007).

Altogether, several mealybugs and at least one soft scale transmit GLRaV-3. This suggests a lack of transmission specificity, which also appears to apply to the other ampelovirus species causing GLD (Tsai et al., 2010; Le Maguet et al., 2012). First instar nymphs appear to be more efficient vectors than adult mealybugs, and transmission likely occurs in a semi-persistent manner. However, these conclusions are based on a limited number of studies, and more research needs to focus on the transmission of GLRaV-3 so that robust knowledge is obtained for the development of science-based disease management strategies that incorporate all aspects of this disease.

### Cytopathology

GLRaV-3 is restricted to the phloem of infected hosts (*V. vinifera*, interspecific hybrids and American rootstocks) in whose organs and tissues it is unevenly distributed (Boscia et al., 1991; Credi and Santucci, 1991; Rowhani et al., 1997). Cytopathological modifications, which are prominent in differentiating sieve tubes, companion cells and phloem parenchyma cells, are characterized by the presence of: (i) inclusion bodies made up of membranous vesicles 50–100 nm in diameter, derived from proliferation of the bounding membrane of mitochondria (Kim et al., 1989). These vesicles, which are released in the cytoplasm following disruption of mitochondria (Faoro et al., 1992), contain a network of fine fibrils identified as RNA, and are thought to be sites of replication (Faoro and Carzaniga, 1995); (ii) loose bundles to compact aggregates of virus particles that often fill the lumen of sieve tubes and may also be localized in the nuclei. Virus clusters can be surrounded by a bounding membrane, giving rise to characteristic intra-cytoplasmic enclaves (Faoro et al., 1992).

### Economic impact of GLRaV-3 and effect on crop and vine health

GLRaV-3 incurs substantial economic losses to the wine, table, raisin, and nursery industries. Yield losses of 20–40% are not uncommon (Habili and Nutter, 1997). The annual cost of GLD is estimated to $1,600–2,350 per hectare of *V. vinifera* cvs. Cabernet Sauvignon and Merlot in New Zealand (Nimmo-Bell, 2006), $300–2,400 per hectare of *V. vinifera* cv. Cabernet Sauvignon in South Africa (Freeborough and Burger, 2008), and $1,000–1,600 per hectare of *V. vinifera* cv. Cabernet Franc in the Finger Lakes region of New York (Atallah et al., 2012).

More specifically, GLRaV-3 reduces yield, cluster size, delays fruit ripening, alters berry color by lowering anthocyanin content, increases titratable acidity, in particular malic and tartaric acids, and changes fruit juice chemistry by reducing soluble solids and modifying aromatic profiles, as shown in *V. vinifera* cvs. Cabernet Franc, Cabernet Sauvignon, Merlot (Borgo et al., 2003), Albariño (Cabaleiro et al., 1999), Chardonnay (Komar et al., 2007), and Dolcetto (Mannini et al., 2012). Wines made from fruits harvested on GLRaV-3-infected cvs. Nebbiolo (Mannini et al., 1998), Tempranillo (Legorburu et al., 2009), and Merlot (Alabi et al., 2012a) have less pigments, phenolics, tannins, and alcohol compared to wines made from healthy vines. In interspecific hybrids Vidal blanc and St Vincent, although GLRaV-3 infection is latent, berry weight is reduced, and titratable acidity is increased in fruit juice (Kovacs et al., 2001).

GLRaV-3 causes a drastic reduction in leaf photosynthesis during post-veraison (Gutha et al., 2012; Mannini et al., 2012) and in free amino acids such as valine and methionine, or glutamic acid in berries of *V. vinifera* cv. Pinot noir (Lee et al., 2009) but an increased skin and pulp weight (Lee and Martin, 2009). Transcriptome analysis showed alteration of the berry maturation process, in particular of genes involved in the anthocyanin biosynthesis and sugar metabolism, in GLRaV-3-infected *V. vinifera* cv. Cabernet Sauvignon (Vega et al., 2011). Similarly, a 2- to 10-fold increase in key genes involved in the flavonoid biosynthetic pathway is measured in leaves of GLRaV-3-infected *V. vinifera* cv. Merlot compared to healthy vines, leading to *de novo* synthesis of anthocyanins such as quercetin and myricetin (Gutha et al., 2010).

A wealth of information is available on the detrimental effects of GLRaV-3 on vine health and crop from field trials with different scion/rootstock combinations or own-rooted vines in Australia, Africa, Europe, and the USA. Though the magnitude of detrimental effects depend on factors such as cultivar, clone, rootstock genotype, vine age, and environmental conditions (Mannini, 2003), data are consistent with GLRaV-3-infected vines being stressed, producing poorly, and substantially reducing vineyard profitability.

### Small RNA profiling in GLRaV-3-infected grapevines

The availability of the grapevine genome sequence allows gene expression profiling, which provides a method to analyze the response of grapevine to various biotic and abiotic stresses at the genetic level. Gene expression in plants is a highly regulated process; one key factor in this regulation are microRNAs (miRNAs), which have been shown to be involved in plant development and plant response to biotic and abiotic stresses. MicroRNAs are a class of small, 21–24-mer, non-coding sRNAs, which are conserved and play a role as “master regulators” of gene expression. In a recent study, Alabi et al. (2012b) profiled endogenous host and viral sRNAs (vsRNAs) in GLRaV-3-infected grapevines by NGS. Altered expression levels of several known *V. vinifera* (vvi)-miRNAs involved in organ and plant development were observed in infected grapes compared to virus-free plants. Particularly vvi-miRNA 156 and 167, which in *Arabidopsis thaliana* target “Squamosa promoter binding protein-like” and “Auxin Response Factor (ARF)” transcription factors, respectively, are both down-regulated, whereas the reverse occurs with vvi-miR166, whose increased levels in *Arabidopsis thaliana* inhibits the expression of its HD-ZIPIII target, thus causing extensive developmental alterations. Surprisingly, a lower expression level of vvi-miR168, which translationally regulates Argonaute 1 (AGO1) expression in *A. thaliana* and *N. benthamiana*, was induced by virus infection. A possible explanation of this unexpected finding may be the condition of the analyzed tissues, which were collected from symptomatic leaves during mid-September, when replicating virus titers are low. Indeed these vines had a low viral RNA concentration, as demonstrated by the small number of vsRNAs detected in the library (0.07% of the total reads). In line with a similar high-throughput analysis performed on citrus plants affected by *Citrus tristeza virus* (CTV) (Ruiz-Ruiz et al., 2011), GLRaV-3-derived sRNAs seem to more densely cover the 3′-terminal region of the viral genome, thus likely originating from the nested set of subgenomic RNAs produced by this virus. The most abundant vsRNAs size class was 21 nucleotides, suggesting that the majority of vsRNAs are processed by a grapevine DCL4-homolog, as previously found for viruses belonging to different taxonomic groups (Pantaleo et al., 2010). Moreover, the involvement of a viral double-stranded RNA as substrate in producing these sRNAs is suggested by the finding of an equal number of vsRNAs of positive and negative polarities. From these investigations, inferences can be drawn which confirm the effects of virus replication on the different small RNA classes observed in annual plants (Chapman et al., 2004; Bazzini et al., 2007). More research in this exciting new branch of disease etiology will shed light on the precise interaction of host plant and virus pathogen.

## Conclusion

Grapevine leafroll disease is considered to be one of the most economically destructive virus diseases of grapevine and a major constraint to the production of premium wine grapes. GLRaV-3 has been more closely associated with GLD than any other GLRaV, supporting the view that it is the “main etiological agent.” Even though the genetic variation observed in the GLRaV-3 genome has been studied more intensively ever since the publishing of the first near complete genome sequence, research on GLRaV-3 lags behind that of other economically important plant viruses. Due to its narrow host range (infecting only *Vitis* species) and being phloem-limited, research on GLRaV-3 has largely focused on epidemiology and the development of reliable detection assays. Phylogenetic studies showed the existence of six genetic variant groups and, with the advances in sequencing technologies, more sequence data will be generated that will indubitably lead to the identification of additional genetic variants. It is therefore necessary to have sensitive and rapid diagnostic methods to test material for GLRaV-3 infection that are able to detect all variants that may influence disease etiology. This implies that newly developed and old diagnostic assays, especially PCR based assays, be verified to be able to detect all genetic variants and continuously be reevaluated to ensure that the assay remains valid as new sequence information becomes available. The role of the different genetic variants of GLRaV-3 in GLD etiology is still largely unknown and elucidating this role is an essential next-step. It is also important to investigate the interactions between the different GLRaV-3 variants, and in combination with the mealybug vectors, to potentially explain the dominant occurrence of some of the genetic variants. The successful construction of an infectious clone of GLRaV-3 provides a platform to study viral replication and gene expression, and determine the function of the GLRaV-3 genes that are currently unknown and also the function of the highly variable extended 5′UTR. GLRaV-3 is one of the most important grapevine viruses and with the use of the latest tools in molecular biology a complete understanding of its role in GLD etiology and host-pathogen interaction is attainable.

## Conflict of Interest Statement

The authors declare that the research was conducted in the absence of any commercial or financial relationships that could be construed as a potential conflict of interest.
